# Exosomes from tubular epithelial cells undergoing epithelial‐to‐mesenchymal transition promote renal fibrosis by M1 macrophage activation

**DOI:** 10.1096/fba.2022-00080

**Published:** 2023-02-06

**Authors:** Yuqing Lu, Rui Zhang, Xiameng Gu, Xuerong Wang, Peipei Xi, Xiaolan Chen

**Affiliations:** ^1^ Affiliated Hospital of Nantong University Nantong China; ^2^ Medical School of Nantong University Nantong China

**Keywords:** epithelial‐to‐mesenchymal transition, exosomes, kidney fibrosis, macrophage, tubular epithelial cells

## Abstract

Kidney fibrosis is the common final pathway of chronic kidney disease (CKD), and it is distinguished by inflammation, mesenchymal transition with myofibroblast formation, and epithelial‐to‐mesenchymal transition (EMT). Macrophages are protuberant inflammatory cells in the kidney, and their roles are dependent on their phenotypes. However, it remains unclear whether tubular epithelial cells (TECs) undergoing EMT can influence the phenotypes of macrophages and the underlying mechanisms during the development of kidney fibrosis. Here, we investigated the characteristics of TECs and macrophages during kidney fibrosis with a focus on EMT and inflammation. We found that the coculture of exosomes from transforming growth factor‐beta (TGF‐β)‐induced TECs with macrophages induced macrophage M1 polarization, while exosomes from TECs without TGF‐β stimulation or stimulation with TGF‐β alone did not induce an increase in M1 macrophage‐related markers. Notably, TECs induced to undergo EMT by TGF‐β treatment released more exosomes than the other groups. Furthermore, it is noteworthy that when we injected exosomes from TECs undergoing EMT into mice, in addition to the high level of inflammatory response and the activation of M1 macrophages, the indicators of EMT and renal fibrosis in mouse kidney tissue were correspondingly elevated. In summary, exosomes from TECs undergoing EMT by TGF‐β treatment induced M1 polarization and led to a positive feedback effect for further EMT and the development of renal fibrosis. Therefore, the obstacle to the release of such exosomes may be a novel therapeutic strategy for CKD.

## Graphical abstract

Our study focuses on the positive feedback loop between renal parenchymal cells and interstitial macrophages and its effect on the overall fibrotic process in the kidney. Results showed that exosomes from TECs undergoing epithelial‐to‐mesenchymal transition (EMT) by TGF‐β treatment induced M1 polarization and led to a positive feedback effect for further EMT and the development of renal fibrosis.

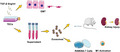

**Abbreviations**
BUN: blood urea nitrogen
CKD: chronic kidney disease
DMEM: Dulbecco's modified Eagle medium
EMT: epithelial‐to‐mesenchymal transition
FBS: fetal bovine serum
PBS: phosphate‐buffered saline
RTECs: renal tubular epithelial cells
TECs: tubular epithelial cells
UUO: unilateral ureteral obstruction

## 1 INTRODUCTION

Chronic kidney disease (CKD) is one of the major causes of early death and is widespread in many countries; as such it has become a major public health concern.^1^ Renal fibrosis occurs via the excessive deposition of an extracellular matrix in the kidney; it is the common terminal pathologic pathway of most CKDs, and it results in end‐stage renal failure.^2^ For this reason, treatments to reduce the associated high morbidity and restore renal function have been investigated, but an optimal treatment has not yet been found.^3^ Renal epithelial‐to‐mesenchymal transition (EMT) is a process in which renal tubular epithelial cells (RTECs) lose epithelial cell markers, such as E‐cadherin or Ksp‐cadherin, and gain mesenchymal cell markers, such as N‐cadherin, fibronectin, and vimentin.^4^ The phenomenon of EMT and its function in renal fibrosis have been observed in many studies,^5^ and as the major constituents of the renal parenchyma, tubular epithelial cells (TECs) are regularly targeted for kidney injury.^6^ There has been recent and increasing interest in the impact of renal intrinsic cells, particularly RTECs, on fibrosis and inflammation. Tubular epithelial cells have been shown not only to be involved in EMT but also to contribute to aggravating inflammation.^7^, ^8^

Exosomes are extracellular vesicles (30–150 nm) that are released by all cell types^9^ and are involved in the crosstalk between nearby and distant cells. As such, their diagnostic value and therapeutic potential are emerging.^10^ Renal tubular epithelial cells secrete exosomes, and a rat model showed that using exosomes from tubular epithelial cells may represent a therapeutic strategy for kidney ischemia–reperfusion injury.^11^ It has also been indicated that the delivery of CCL2 mRNA from tubular epithelial cell exosomes to macrophages leads to severe kidney inflammation, which suggests that exosomes derived from TECs could serve as a new therapeutic target.^12^ However, whether TECs undergoing EMT can influence the phenotype and underlying mechanisms of macrophages remains unclear.

A recent study using a mouse model of renal ischemia/reperfusion injury indicated that tubular epithelial cells can phenotypically transform macrophages via exosome mediation,^13^ which suggested that exosomes secreted by TECs in pathological states can mediate the exchange of information between renal intrinsic cells and renal interstitial inflammatory cells. Inspired by this discovery, we explored whether TECs (in which EMT occurs) can influence the activation state and phenotype of macrophages and the underlying mechanisms. Our study focuses on the positive feedback loop between renal parenchymal cells and interstitial macrophages and its effect on the overall fibrotic process in the kidney.

## 2 MATERIALS AND METHODS

### 2.1 Animal models

The mice provided by the Experimental Animal Center of Medical College of Nantong University were housed in groups of five per plastic cage on sawdust bedding in a 12/12 light–dark cycle (light‐on period, 06:00–18:00) at the temperature of around 22°C. They were fed a standard diet and provided access to filtered water ad libitum. Renal fibrosis was established in unilateral ureteral obstruction (UUO)‐induced mice as described previously. Briefly, the mice were anesthetized with pentobarbital sodium. The right ureter was completely ligated with a fine suture material (4–0 silk) at two points after exposure. Male BALB/c mice (6–8 weeks old) were injected with exosomes manufactured in vitro from 8 × 10^6^ TECs with or without TGF‐β (5 ng/ml) treatment through tail vein every day, in a total volume of 100 μl, or equal volumes of sterile phosphate‐buffered saline (PBS), five mice for each group. And, the mice were euthanized 72 h after the last injection (Day 7).^12^ Blood and kidney tissues were collected for later analysis both in Day 7. All animal experiments were approved by the Institutional Animal Care and Use Committee of Nantong University following the current guidelines for animal care and welfare.^14^

### 2.2 Cell culture

RAW 264.7 cells (donated by Dr. Bin, Yang, University of Leicester) were maintained in Dulbecco's modified Eagle medium (DMEM) containing 1% l‐glutamine, streptomycin (100 μg/ml), penicillin (100 units/ml), and 10% fetal bovine serum (FBS). Mouse renal tubular cell line TCMK‐1 (donated by Dr. Tianyi Pan of Zhongshan Hospital [affiliated with Fudan University]; CCL‐139) was used to explore tubular epithelial cells (TEC) functions in vitro. Tubular epithelial cells were grown in Dulbecco's modified Eagle's medium (DMEM)/F‐12 medium (Gibco, Carlsbad, USA) with 10% (v/v) fetal calf serum (Gibco), 2 mM l‐glutamine (Gibco), 100 U/ml penicillin G, and 100 mg/m{Fujiki, 2019 #1}l streptomycin (Sigma) at 37°C in a 5% CO_2_ humidified atmosphere. Tubular epithelial cells (1 × 10^5^/well) were stimulated with 5 ng/ml transforming growth factor‐beta (TGF‐β) for 48 h for further research, after seeding in 6‐well plates.

### 2.3 Serum creatinine and blood urea nitrogen

Serum was harvested to detect the concentration of blood urea nitrogen (BUN) using a kit (Stanbio Laboratory, United States) as per the manufacturer's protocol. A Jaffe reaction‐based kit (Stanbio Laboratory) was used to assess serum levels of creatinine (Scr) levels.

### 2.4 Periodic acid–Schiff staining and masson staining

After removal, the mouse kidney was fixed with 10% formalin for 24 h at 4°C and then entrenched in paraffin. Sections were cut at 5 μm and incubated with 0.5% periodic acid solution for 20 min, followed by staining with Schiff's reagent for 30 min. After washing with tap water for 15 min, a light microscope was used to observe the sections.

For Masson Staining, after dewaxing, the paraffin sections were washed with distilled water. Nuclei were stained with Weigert sappan semen for 10 min. The sections were then washed with water, dyed with Masson Ponceau acid red solution for 10 min, and then fractionated with 1% molybdenum phosphate solution in 2% glacial acetic acid for 3–5 min after immersion for 10 min. Subsequently, the sections were colored with aniline blue for 5 min without washing, and they were then immersed in a 0.2% glacial acetic acid solution for 1 min. Finally, the sections were dehydrated using anhydrous alcohol (xylene for transparency, neutral glue for sealing).

### 2.5 Immunohistochemical analysis and immunofluorescence staining

Sections were incubated overnight at 4°C with a primary antibody against CD86 (1:1000; Abcam), followed by incubation for 30 min at room temperature with a secondary antibody (40 μl; Abcam). Images were captured using a light microscope.

Immunostaining was performed on frozen mouse tissue sections fixed in 2% paraformaldehyde following a previously described protocol.^15^ In brief, sections were labeled overnight with different combinations of directly conjugated primary antibodies as follows: FITC‐conjugated anti‐F4/80 antibody (Bioss); FITC‐conjugated anti‐iNOS (eBioscience); and Cy3‐conjugated α‐SMA antibody (Sigma), or E‐cadherin (Proteintech) conjugated with Pacific Blue (P30013; Invitrogen), or PE‐conjugated Pou4f1 (sc‐8429 PE; Santa Cruz). Sections were washed, and DNA was counterstained with 4′,6‐diamidino‐2‐phenylindole (DAPI), followed by observation under a fluorescence microscope (Carl Zeiss Axio Observer Z1). Trainable Weka Segmentation, ROI Manager, Cell Counter, and some powerful Plugins of FIJI ImageJ were used to complete semi‐quantitative analysis.^16^, ^17^

### 2.6 Exosome extraction and identification and Western blot

After TECs were maintained with 10% exosome‐removing FBS (Beyotime, China) for 24 h, supernatants were collected and centrifuged at 300 *g* for 5 min, 3000 *g* for 15 min, and 15,000 *g* for 30 min at 4°C. After filtration with a 0.22‐mm filter (Millipore), the supernatant was further ultracentrifuged at 100,000 *g* for 2 h at 4°C and ultracentrifuged at 100,000 *g* for 2 h again after washing with PBS. Finally, 100 μl PBS was used to resuspend the pellets for further study. The double‐layer capsule ultrastructure of exosomes was observed using a transmission electron microscope (JEM‐2100F). When TECs grew to 70%–80% confluence, PKH67 dye (Sigma) was used (1 × 10^7^ cells stained with 2 × 10^−6^ M PKH67) to stain the cells at 37 degrees for 30 min. After staining stopped by buffer with 5% albumin, the free dye was washed away with PBS for three times. Tubular epithelial cells were then stimulated with TGF‐β (5 ng/ml), for 48 h, or with PBS as a control. The exosomes were washed completely in 20 ml of PBS and collected by ultracentrifugation as described above, and was added to RAW264.7 for 24 h. And, the most common cause of fuel residues is inadequate washing of the cells. So, buffer with 5% albumin was used to stop staining and we washed cells three to five times after labeling and transfer samples to new tubes between washes. The exosomes were washed completely in 20 ml of PBS and collected by ultracentrifugation as described above, and was added to RAW264.7 for 24 h. A confocal microscope (Olympus, Japan) was used to visualize these labeled exosomes after incubation with macrophages for 24 h. In addition, NTA was used to measure the particle size and concentration of purified exosomes diluted with PBS buffer using ZetaView (Particle Metrix). The data were analyzed using the ZetaView 8.

For the Western blot tests, cells and exosomes were harvested to extract proteins using RIPA lysis buffer (Beyotime) with protease inhibitors (Beyotime), the cells and exosomes were harvested to extract proteins. A Nanodrop ultra‐micro spectrophotometer was used to measure total proteins, followed by equal protein loading and division, and then transferred onto a polyvinylidene fluoride membrane (Millipore). After blocking with 5% skimmed milk for 2 h at room temperature, the membranes were incubated with primary antibodies against E‐cadherin (1:1000; Cell Signaling Technology), α‐SMA (1:1000; Abcam), vimentin (1:10,000; Proteintech), N‐cadherin (1:2000; Proteintech), iNOS (1:1000; Cell Signaling Technology), HSP70 (1:2000; Proteintech), TSG101 (1:2000; Proteintech), and CD63 (1:1000; Abcam) at 4°C overnight and then incubated for 2 h with a corresponding secondary antibody (1:5000; Absin) for 2 h. Finally, a chemiluminescent detection system (Bio‐Rad) was used to detect the blots, and tubulin (1:5000; Abcam) and β‐actin (1:5000; Abcam) were used as controls.

### 2.7 Polymerase chain reaction (PCR)

Quantitative real‐time polymerase chain reaction isolater (Vazyme) was used to extract total RNA from cells or kidney tissue RNA following the manufacturer's protocol. The concentration and purity of the RNA samples were measured using a nanodrop ultra‐micro spectrophotometer. The RNA was reverse transcribed into cDNA using HiScript III RT SuperMix for qPCR (Vazyme). The PCR was performed on a real‐time PCR system (Bio‐Rad) using ChamQ Universal SYBR qPCR Master Mix (Vazyme). The data were normalized to those of β‐actin. The following primer sequences were used: TNF‐α (mouse): forward 5′‐CATCTTCTCAAAATTCGAGTGACAA‐3′, MCP‐1 (mouse): forward 5′‐CTTCTGGGCCTGCTGTTCA‐3′, reverse 5′‐CCAGCCTACTCATTGGGATCA‐3′, reverse 5′‐TGGGAGTAGACAAGGTACAACCC‐3′; IL‐1β (mouse): forward 5′‐TGGGAGTAGACAAGGTACAACCC‐3′ and reverse 5′‐AAGGTCCACGGGAAAGACAC‐3; IL‐10 (mouse): forward 5′‐ CTTACTGACTGGCATGAGGATCA‐3′ and reverse 5′‐ GCAGCTCTAGGAGCATGTGG‐3′; Snail (mouse): forward 5′‐ CTGCTTCGAGCCATAGAACTAAAG‐3′ and reverse 5′‐GAGGGGAACTATTGCATAGTCTGT‐3′; E‐cadherin (mouse): forward 5′‐CAGCCTTCTTTTCGGAAGACT‐3′ and reverse 5′‐GGTAGACAGCTCCCTATGACTG‐3′; vimentin (mouse): forward 5′‐CGGAAAGTGGAATCCTTGCA‐3′ and reverse 5′‐CACATCGATCTGGACATGCTG‐3′; and Fibroblast‐specific protein 1 (FSP‐1) (mouse): forward 5′‐GATGAGCAACTTGGACAGCA‐3′ and reverse 5′‐ATGTGCGAAGCCAGAGT‐3′. Samples were analyzed in triplicates.

### 2.8 Wound‐healing assay and enzyme‐linked immunosorbent assay (ELISA)

The RAW264.7 cells monolayer was scraped in straight lines using a pipette tip, and it was then gently washed to remove detached cells and replenished with a fresh medium. Following incubation for 24 h, the wounds were observed by phase‐contrast microscopy. The data were analyzed using ImageJ software.

The levels of TNF‐α, IL‐6, and IL‐1β in the supernatant of Raw264.7 were determined using a commercial ELISA kit (eBioscience) according to the recommended protocol.^18^

### 2.9 Statistical analysis

The experimental data were analyzed using Prism 8.0 GraphPad Software. All experiments were repeated at least three times, with three replicate wells per design, and data are shown as mean ± standard deviation. Statistical analyses were performed using *t*‐test or one‐way ANOVA

## 3 RESULTS

### 3.1 Activation of M1 macrophage with EMT in the unilateral ureteral obstruction‐induced CKD model

**FIGURE 1:**
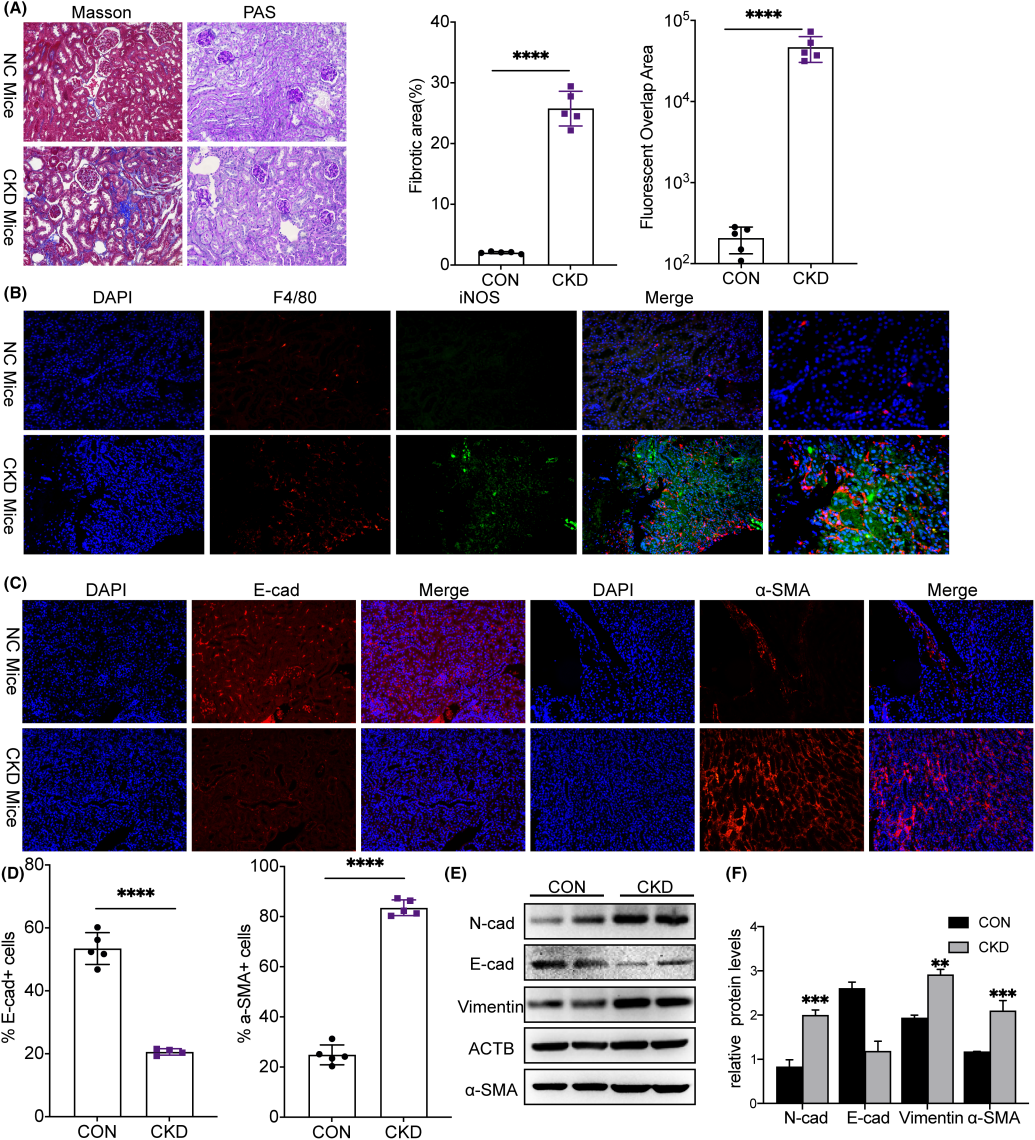
M1 Macrophage activated with EMT in the unilateral ureteral obstruction (UUO)‐induced CKD Model. Mice were assembled and subjected to UUO. (A) Kidney tissue was harvested on Day 7 after the operation. PAS staining and Masson staining images of kidneys from UUO‐injured or sham mice (original magnification ×200), including representative data of histologic (Masson) changes. (B) Representative immunofluorescent staining of kidney tissues from NC and CKD groups showing the simultaneous expression of F4/80 and iNOS, and the semi‐quantitative analysis of fluorescent overlap area. (C, D) Representative IHC images of α‐SMA and E‐cadherin in the kidneys (original magnification ×400). (E) The protein expressions of EMT‐related markers and fibrosis‐related markers, including vimentin, E‐cadherin, N‐cadherin, and α‐SMA, were determined using Western blotting, including the quantitative analysis (F). ****p < 0.0001, ***p < 0.001 versus sham mice. UUO, unilateral ureteral obstruction. *n* = 5 for each group of mice.

We first evaluated the M1‐activation levels of macrophages and the occurrence level of EMT in the mouse model of UUO‐induced renal fibrosis and then conducted periodic Acid–Schiff (PAS) and Masson staining to verify the success of our mouse model. The results showed that UUO caused huge ECM deposition in renal tissues and tubular injury occurred (Figure 1A). The increase in fluorescent overlap area in CKD mice was verified by immunofluorescence staining, and the results suggested the enhanced activation of M1‐type macrophages (Figure 1B). Given the importance of EMT in the development of renal fibrosis, immunofluorescent staining was further conducted to examine the levels of stromal cell markers and epithelial cell adhesion factor (Figure 1C,D). The results of Western blotting revealed that the expression of α‐SMA was increased in the epithelial cells and E‐cadherin was decreased, which further confirmed the enhanced EMT in the CKD group. The results showed that, compared with the control group, the levels of α‐SMA, vimentin, and N‐cadherin were increased, and the protein levels of E‐cadherin in the CKD group were significantly reduced (Figure 1E,F). These results imply that UUO treatment induced renal fibrosis, the occurrence of EMT, and increased the level of expression of M1‐activation macrophages.

### 3.2 TGF‐β induces an EMT‐like process in cultured TECs

**FIGURE 2:**
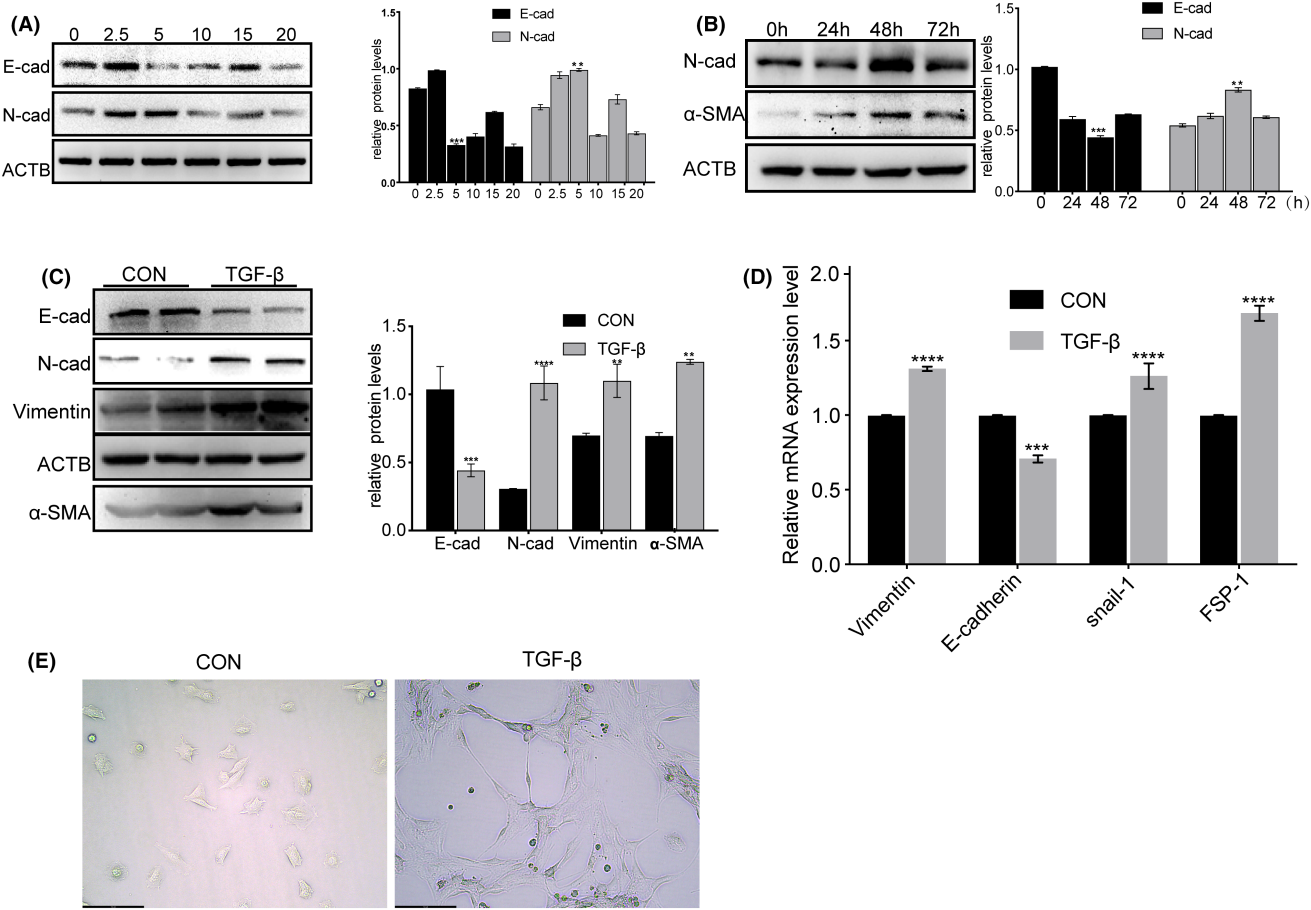
EMT process resulted from TGF‐β in cultured TECs. TGF‐β was used to induce TECs EMT model in vitro. (A) Protein expression of TECs after stimulation for 24 h with different concentrations of TGF‐β. (B) Protein expression of TECs stimulated by 5 ng/ml of TGF‐β for different times. (C) Amount of EMT‐related marker proteins expressed by TECs stimulated at optimal concentration and time. (D) TECs were treated with 5 mg/ml TGF‐β for 48 h, and the mRNA expressions of vimentin, E‐cadherin, Snail‐1, and FSP‐1 in TECs were then measured by real‐time PCR. ****p < 0.0001, ***p < 0.001, **p < 0.01 versus control tubular epithelial cells. (E) Morphology of TECs with or without TGF‐β stimulation. CON, control tubular epithelial cells; TGF‐β, tubular epithelial cells with TGF‐β stimulation (5 ng/ml, 48 h).

TGF‐β was used to stimulate RETCs to establish an EMT cell model in vitro, and the protein expression levels of E‐cadherin and N‐cadherin were used as indicators to map the optimal concentration and time of TGF‐β stimulation for EMT to occur in TECs. As seen in Figure 2A,B, the relative expression of E‐cadherin and N‐cadherin was the lowest and highest, respectively, at a TGF‐β concentration of 5 ng/ml and a stimulation time of 48 h. To further test whether 5 ng/ml stimulation for 48 h was the optimal stimulation condition, we used Western blotting to examine the expression of epithelial cell marker proteins (Figure 2C). The results demonstrated that under these conditions, the expressions of α‐SMA, N‐cadherin, and vimentin proteins increased, whereas that of E‐cadherin protein decreased. In addition, qPCR results showed that the expressions of EMT‐related transcription factors, including Snail‐1 and FSP‐1, were also increased (Figure 2D). In addition, TECs treated with 5 ng/ml TGF‐β for 48 h induced EMT‐like morphological changes. In brief, cell morphology changed from epithelial‐to‐fibroblastic‐like spindle shape (Figure 2E). Taken together, these results verified that the most pronounced EMT occurred in mouse RTECs after treatment with 5 ng/ml TGF‐β for 48 h.

### 3.3 TGF‐β‐treated cells undergoing EMT secrete more exosomes than NC‐treated TECs

**FIGURE 3:**
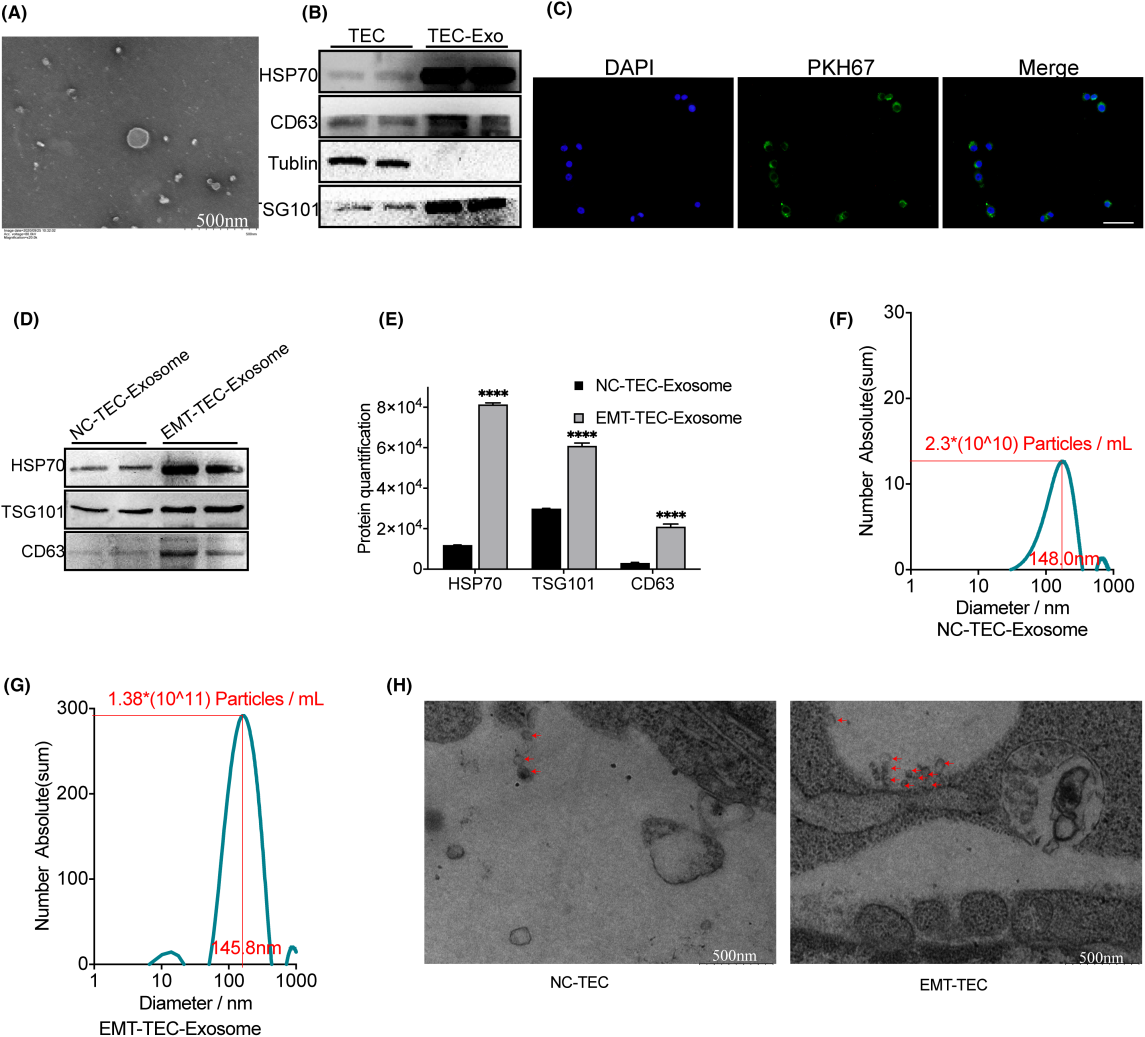
Secretion of exosomes by TECs under different stimulations. (A) Representative transmission electron microscopy (TEM) images of exosomes derived from TECs. (B) Exosomes excreted were confirmed and quantified by Western blotting for TSG101, CD63, and HSP70 exosome markers. (C) EVs isolated from PKH67‐labeled TECs were suppressed into RAW264.7 cells (original magnification ×200). (D) Exosomes from the NC‐TECs and EMT‐TECs expressed different amounts of exosomal marker proteins, including the quantitative analysis (E). Concentration and size distribution determined by Nanoparticle tracking analysis (NTA) in the NC‐TEC‐Exosome group (F) and the EMT‐TEC‐Exosome group (G). (H) Electron micrographs of secreted exosomes from TECs with and without TGF‐β induction (exosomes are shown with arrows, bar = 500 nm). ****p < 0.0001 versus the NC‐TEC‐Exosome. EMT‐TEC, TECs undergoing EMT; EMT‐TEC‐Exosome, exosomes from tubular epithelial cells induced epithelial‐to‐mesenchymal transition; NC‐TEC, natural control TECs group; NC‐TEC‐Exosome, exosomes from normal tubular epithelial cells.

Through the transfer of their cargo, exosomes mediate intercellular communication during the development of renal fibrosis. In this study, exosomes were separated from the cell culture supernatant of TECs by high‐speed centrifugation, and transmission electron microscopy (TEM) was conducted (Figure 3A). Detection of the levels of exosome surface markers by Western blotting verified the purity of exosomes (enrichment of marker proteins including HSP70, CD63, and TSG‐101 were detected in the purified exosomes, without the expression of tubulin). The results showed that the exosome preparations were not contaminated with cells (Figure 3B). In addition, to determine whether the exosomes from TECs can be ingested by RAW264.7, the exosomes were labeled with fluorescent dye PKH67 followed by co‐incubation with RAW264.7 cells for 24 h. The results showed that RAW264.7 cells ingested the exosomes derived from TECs (Figure 3C). Then, exosomes were isolated from the cell culture supernatants of the same number of NC‐treated TECs and TGF‐β‐treated TECs and detected by Western blotting. Notably, the expression of exosomal marker proteins was higher in exosomes from TGF‐β‐treated cells undergoing EMT than in NG‐treated TECs, which indicated that TECs undergoing EMT may release greater numbers of exosomes (Figure 3D). Furthermore, Nanoparticle tracking analysis (NTA) confirmed the increased number of the exosomes in the supernatants of tubular epithelial cells (2.3 × 10^10^ and 1.3 × 10^11^ particles/ml for normal TECs and EMT‐TECs, respectively) (Figure 3E,F). In addition, electron micrographs of secreted exosomes from TECs with and without TGF‐β induction were performed (Figure 3H). As shown in the photographs, TECs can secrete more exosomes under TGF‐β induction (5 ng/48 h) (exosomes are shown with arrows, bar = 500 nm). These results establish the premise that TGF‐β‐treated TECs can secrete more exosomes than NG‐treated TECs, and these are then ingested by macrophages.

### 3.4 Exosomes derived from EMT‐TECs triggered M1 macrophage activation in vitro

**FIGURE 4:**
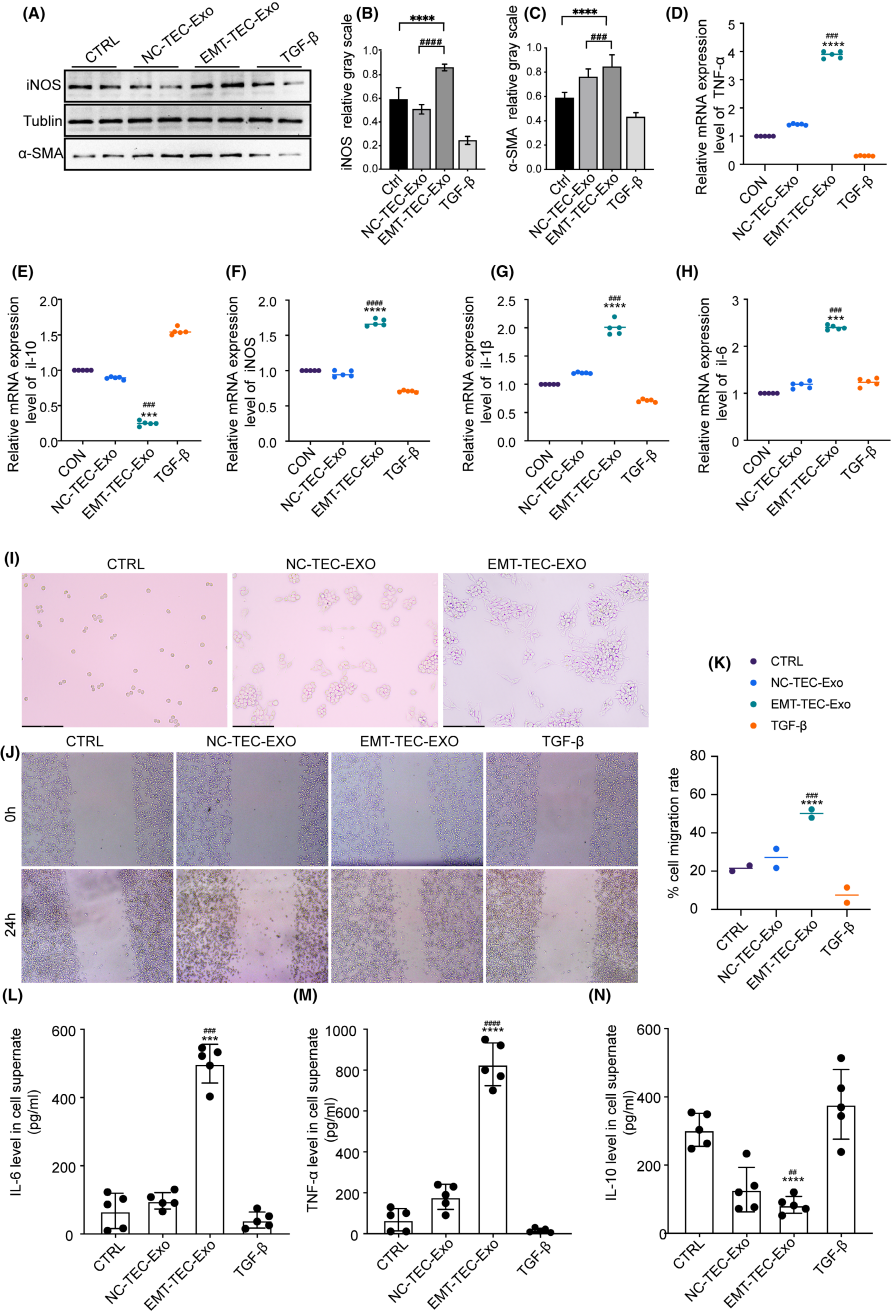
Exosomes derived from EMT‐TECs triggered M1 macrophage activation in vitro. RAW264.7 cells were treated with exosomes from TECs for 24 h. (A) The image produced by Western blot assay and the column diagram were calculated from the relative grayscale of iNOS (B) and α‐SMA (C) stripe. (D–H) the expressions of TNF‐α, IL‐10, iNOS, IL‐1β, and IL‐6 in cells were then measured by qRT‐PCR. (I) The morphology of TECs in different groups. (Original magnification ×200) (J, K). Wound‐healing assays and cell migration rates. (L) IL‐6, (M) TNF‐α, (N) IL‐10 in the macrophage supernatant measured using ELISA. Data are presented as mean ± SD for three independent experiments. ****p < 0.0001, ***p < 0.001 versus the CTRL group, ^####^ *p* < 0.0001, ^###^ *p* < 0.001, ^##^ *p* < 0.01 versus the NC‐TEC‐Exo group. CTRL, normal RAW264.7; NC‐TEC‐Exo, RAW264.7 stimulated by the exosomes from common tubular epithelial cells; EMT‐TEC‐Exo, RAW264.7 stimulated by the exosomes from tubular epithelial cells undergoing epithelial‐to‐mesenchymal transition; TGF‐β, RAW264.7 treated by TGF‐β.

As shown in Figure 4A, EMT‐TEC‐Exo increased the expression levels of the M1 macrophage marker iNOS (qualificative analysis in Figure 4B) and anti‐α smooth muscle actin (α‐SMA) (qualificative analysis in Figure 4C), which is a marker of fibrosis. The mRNA expression of macrophage cell M1‐activation markers, such as TNF‐a, IL‐1b, Il‐6, and iNOS, was higher in the EMT‐TECs‐Exo group than in the NC‐TECs‐Exo group (Figure 4D,F–H), whereas the trend for IL‐10 is the opposite of the aforementioned (Figure 4E).

Exosomes from various TEC groups affected the morphology of RAW264.7 macrophages. EMT‐TEC‐Exo treatment triggered deviations in cell morphology (Figure 4I). The EMT‐TEC‐Exo‐treated cells were attached, larger, and formed long, slim, pseudopodia‐like protrusions, whereas the control cells were confluent and round after 24 h. In the wound‐healing assay, compared with macrophages in NC‐TEC‐EXO group, EMT‐TECs‐Exo‐treated macrophages indicated considerably increased migration at a rate of approximately two times that of untreated macrophages (Figure 4J,K). Using ELISA, we found that the levels of TNF and IL‐6 increased (Figure 4L,M), while the level of IL‐10 decreased correspondingly in the cell culture supernatant of the EMT‐TEC‐Exo group (Figure 4N).

### 3.5 Influence of exosomes released from TECs undergoing EMT in vivo

**FIGURE 5:**
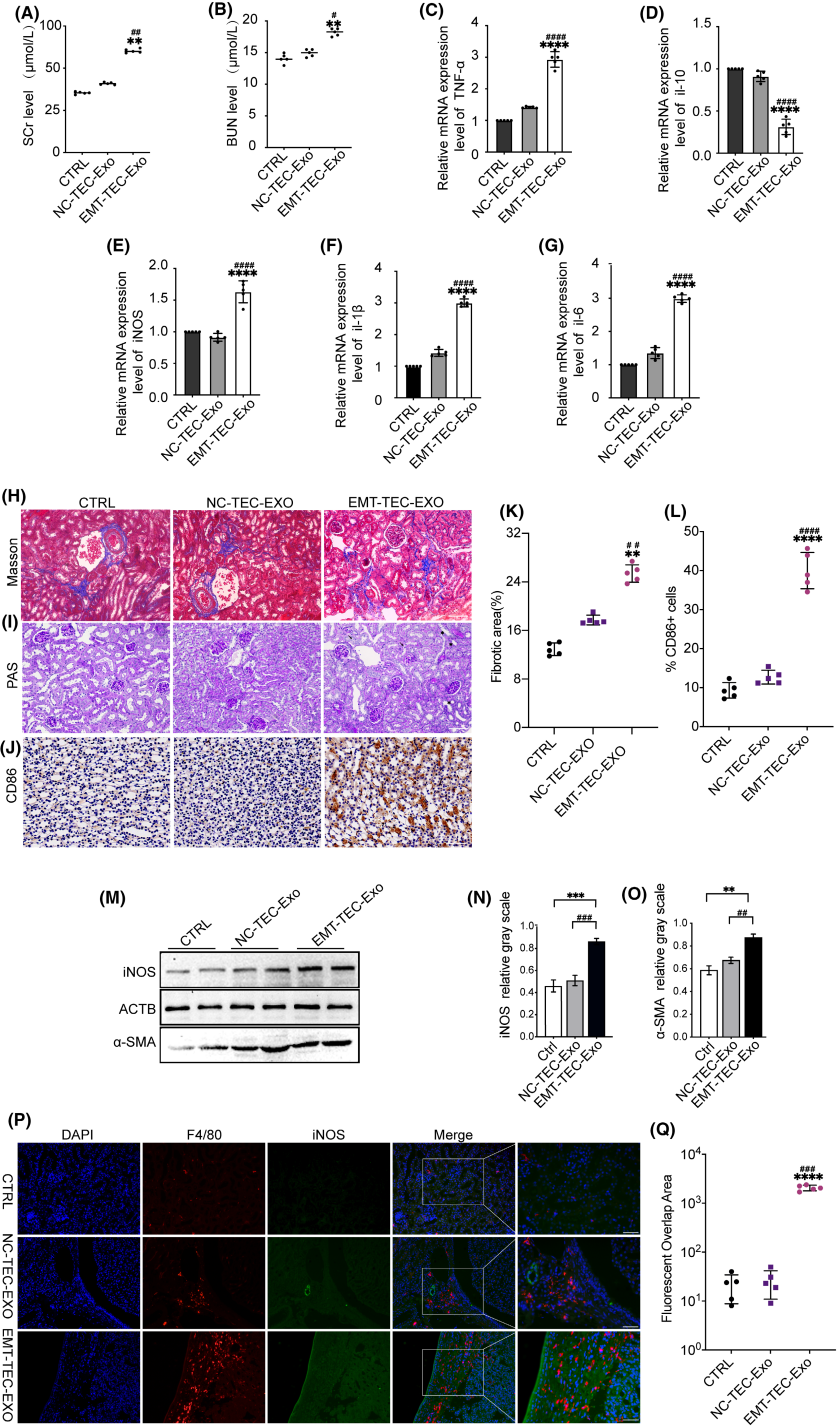
Exosomes from TECs undergoing EMT led to renal fibrosis and M1 macrophage activation. Mice injected exosomes secreted by TECs undergoing EMT or not show different renal functions. (A) Serum SCr and (B) BUN levels in different groups. (C–G) mRNA expression levels of macrophage phenotype‐related marker genes (TNF‐α, IL‐10, and iNOS) and inflammatory factors (IL‐1β and IL‐6) in the injured kidney as assessed by real‐time PCR. Photomicrographs illustrating Masson trichrome staining (H) and PAS staining (I) of kidney tissue from mice in various treatment groups; Note that brush border loss (*) and epithelial cell effacement (↓) were present (original magnification ×200). (K) Quantification of Masson trichrome staining results. (J) Representative data of immunohistologic changes (CD86 immunostaining) in mice. (L) Quantitative analysis for the CD86–positive cells. (M) The levels of iNOS and α‐SMA were measured by Western blotting and the column diagram were calculated from the relative grayscale of iNOS (N) and α‐SMA (O) stripe. (P) Representative immunofluorescent staining of F4/80 (red) and iNOS (green) in the kidney tissues from different groups (original magnification ×200). (Q) Calculate the area of F4/80‐positive and iNOS‐positive overlapping areas to quantify the fluorescence results. ****p < 0.0001, ***p < 0.001, **p < 0.01 versus the CTRL group, ^####^ *p* < 0.0001, ^###^ *p* < 0.001, ^##^ *p* < 0.01 versus the NC‐TEC‐Exo group. CTRL, control mice; EMT‐TEC‐Exo, mice injected with exosomes produced by TEC after the EMT; NC‐TEC‐Exo, mice injected with exosomes from normal TECs by tail vein, *n* = 5

In this study, to validate the role of exosomes produced by TECs that underwent EMT on macrophage polarization, we established mouse models by injecting the tail vein with exosomes from TECs using various treatments. We observed a high level of SCr (Figure 5A). and BUN (Figure 5B). in mice that received an EMT‐TECs‐Exosome (exosomes from TECs undergoing EMT) injection regularly, whereas there were no novel changes in the NC‐TECs‐Exo group (mice injected exosomes from TECs without undergoing EMT). The real‐time PCR analysis revealed that the EMT‐TEC‐Exosome injection caused a threefold increase in TNF‐α, a decrease in IL‐10, and a threefold increase in IL‐1β compared with the NC‐TECs‐Exo group. The biomarkers of M1 macrophages and the mRNA expression levels of iNOS indicated that M1 macrophage activation was linked to the state of TECs (Figure 5C–G). The tubular injury occurred in the EMT‐TEC‐Exo group and was characterized by epithelial cell effacement and brush border loss (Figure 5I). Immunostaining of markers of M1‐type activation macrophages (CD86) revealed positive staining in interstitial areas of the EMT‐TEC‐Exo group, which indicated M1‐type activation and inflammatory infiltration of macrophages (Figure 5J,L). Hence, an advanced protein level of iNOS and α‐SMA was observed in the kidneys after exosome‐EMT treatment (Figure 5M–O).

We determined the function of EMT‐TECs‐Exo in vivo. Furthermore, double immunofluorescence staining demonstrated colocalization between F4/80 and iNOS staining, and this revealed the aggregation of M1‐type macrophages (Figure 5P,Q). These results suggest that TECs undergoing EMT lead to kidney injury by transporting exosomal messages into naive macrophages to trigger activation.

**FIGURE 6:**
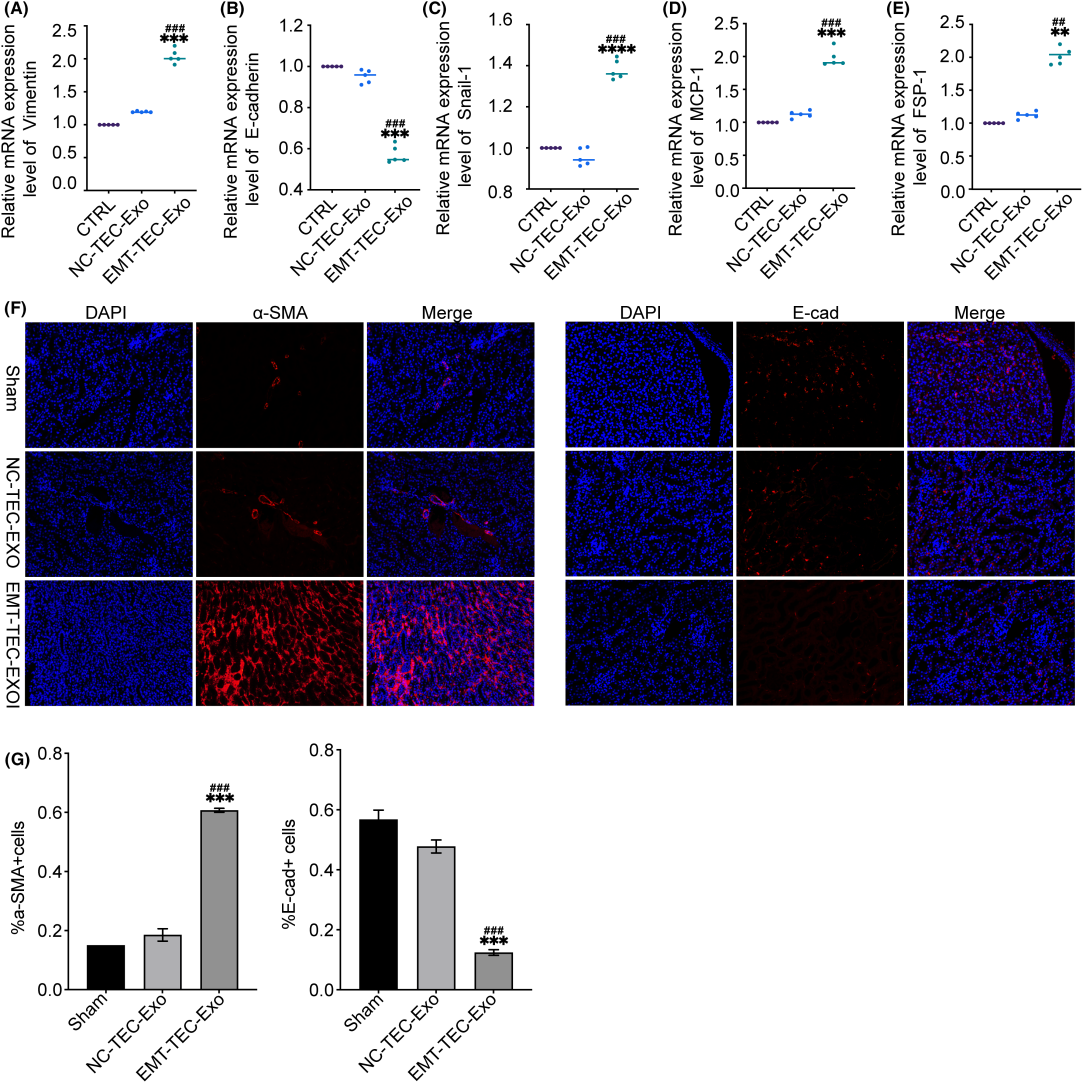
EMT‐TEC‐Exo induced an EMT‐like process in vivo. (A–E) The mRNA expressions of vimentin, E‐cadherin, Snail‐1, MCP‐1, and FSP‐1 in the exosome‐injected kidneys were determined by real‐time PCR. (F–G) Representative images of α‐SMA and E‐cadherin in renal tissues from different groups were detected by IF (original magnification: ×200) and the semi‐quantitative analysis of the number of α‐SMA or E‐cadherin positive cells. ****p < 0.0001, ***p < 0.001, **p < 0.01 versus the CTRL group, ^####^ *p* < 0.0001, ^###^ *p* < 0.001, ^##^ *p* < 0.01 versus the NC‐TEC‐Exo group. CTRL, control mice; EMT‐TEC‐Exo, mice injected with exosomes produced by TEC after the EMT; NC‐TEC‐Exo, mice injected with exosomes from normal TECs by tail vein, *n* = 5

Interestingly, the mRNA expressions of Snail‐1, vimentin, MCP‐1, and FSP‐1 were also significantly upregulated in mice treated with EMT‐TEC‐Exo compared with the sham and NC‐TEC‐Exo groups. Correspondingly, a lower mRNA level of E‐cadherin was detected in the kidney tissue with the EMT‐TEC‐Exo treatment (Figure 6A–E). Moreover, the immunofluorescence results demonstrated that the macrophages simulated by EMT‐TEC‐Exo contained a lower level of E‐cadherin than other groups, followed by a threefold increase in the expression of α‐SMA (Figure 6F,G).

## 4 DISCUSSION

Epithelial‐to‐mesenchymal manifests with a loss of epithelial cell features (to varying degrees) and the acquisition of mesenchymal cell features, and its role in the progression of renal fibrosis cannot, therefore, be underestimated. Similarly, macrophages are key regulators of the onset and progression of renal fibrosis.^19^, ^20^ However, how RTECs influence macrophage activation in the context of renal fibrosis progression has not yet been determined. Through in vitro and in vivo studies, we found that RTECs that develop EMT produce more exosomes, and via exosomes, they achieve a cellular scramble with macrophages. This causes M1‐type activation of macrophages and the secretion of pro‐inflammatory factors and chemokines, which further aggravate renal fibrosis in CKD.

In our study, a renal fibrosis model was established with UUO, and the success of the model was verified by H&E staining and Masson staining. We found that in UUO‐induced CKD mice, the epithelial cell marker expression decreased, the mesenchymal cell marker expression increased, and EMT occurred. At the same time, macrophage aggregation increased, and the markers of M1‐type macrophages were highly expressed. In addition, in vitro TGF‐β treatment‐induced EMT in TECs, along with increased marker protein expression of exosomes in the supernatant of TECs, suggested that more exosomes were released from TECs that underwent EMT, and that tubular‐derived exosomes could be taken up by macrophages. As we observed enhanced EMT in the renal tubular epithelial cells of CKD mice accompanied by M 1‐type polarization of renal interstitial macrophages, we hypothesized that exosomes produced by TECs could be taken up by macrophages and promote M1‐type polarization of macrophages, and this hypothesis was tested in detail using in vitro cell culture models and in vivo assays.

In vitro, we used cellular models of TECs to establish EMT, and the TECs in the EMT state were found to secrete more exosomes; this result agreed with that of a previous study.^13^ Subsequently, we found increased expressions of IL‐6, IL‐1b, MCP‐1 (a pro‐inflammatory cytokine), the increased expression of M1 surface markers, and an increased M1/M2 ratio, and M1 polarization was promoted in macrophages that ingested EMT‐TECs‐Exo compared with direct TGF‐β stimulation and co‐culture with NC‐TECs‐Exo. Furthermore, our in vitro studies further confirmed that in addition to the phenotypic changes, the cells had an enhanced ability to migrate and secrete chemokines and pro‐inflammatory factors. Thus, we suggest that TEC‐derived exosomes may characterize a new courier for cell–cell communication during renal injury.

To determine the role of EMT‐TECs‐Exo in vivo, we isolated exosomes from TECs that underwent EMT and then applied them to mice via tail vein injection. Severe renal injury and the upregulation of pro‐inflammatory factors were then observed. By contrast, equivalent amounts of NC‐TECs‐Exo were used in the kidney, after which there was no evidence of M1 macrophage triggering, and renal injury was not promoted. This thus suggests that the exosomes secreted by TECs in which EMT occurs may be an important mediator of macrophage M1 type activation and further renal injury.

Interestingly, we found that exosomes derived from TECs undergoing EMT could also lead to the loss of epithelial cell markers and the acquisition of mesenchymal cell markers in vivo, as shown in Figure 6. These data demonstrate that a positive feedback loop may exist between exosomes from EMT‐TECs and M1 macrophage activation. If this loop works as predicted, disrupting this regulation would incompletely chunk the process of fibrosis, and this could be an excellent novel treatment strategy for fibrosis. In future studies, we will further explore whether this regulation is dependent on a certain cytokine or micro‐RNA, as well as other important components in this positive feedback closure loop, and as such we will extend our in‐depth study of fibrosis mechanisms. A very recent study found that inhibiting exosomal secretion in PTECs by knocking out *Rab27a* (a key exosome regulatory gene) could inhibit the excessive inflammatory response in PTECs through the miR‐26a‐5p/CHAC1/NF‐kB pathway, thereby delaying the progression of diabetic kidney disease,^21^ and another study reported similar findings, that inhibiting IRF‐1/Rab27a mediated exosome secretion accelerated albumin degradation, which could reverse tubule injury with albumin overload, and alleviate tubular inflammation by suppressing lysosomal degradation.^22^ These findings suggest that targeting exosome secretion would be a novel therapeutic strategy for CKD associated with various causes.

In summary, our study showed that TGF‐β treatment induced EMT and increased exosome release from renal tubular epithelial cells; this can induce M1‐type activation of macrophages and promote the scattering of renal parenchymal and interstitial cells.

## AUTHOR CONTRIBUTIONS

Xiaolan Chen designed, supervised, and revised the manuscript. Yuqing Lu and Rui Zhang performed the experiments and wrote the manuscript. Xuerong Wang, Peipei Xi, and Xiameng Gu analyzed the data.

## FUNDING INFORMATION

This study was supported by the Science and Technology Project of Nantong City (MS22020009).

## CONFLICT OF INTEREST

The authors affirm that the research was directed in the absence of any commercial or financial relationships that could be construed as a potential conflict of interest.

## Supporting information


Appendix S1

## Data Availability

The data supporting this study's findings are available from the corresponding author or Yuqing Lu, upon reasonable request.
